# Increasing Working Memory Load Reduces Processing of Cross-Modal Task-Irrelevant Stimuli Even after Controlling for Task Difficulty and Executive Capacity

**DOI:** 10.3389/fnhum.2016.00380

**Published:** 2016-08-03

**Authors:** Sharon S. Simon, Erich S. Tusch, Phillip J. Holcomb, Kirk R. Daffner

**Affiliations:** ^1^Division of Cognitive and Behavioral Neurology, Department of Neurology, Center for Brain/Mind Medicine – Brigham and Women’s Hospital, Harvard Medical School, BostonMA, USA; ^2^Old Age Research Group (PROTER), Institute of Psychiatry, University of São Paulo School of MedicineSão Paulo, Brazil; ^3^Department of Psychology, Tufts University, MedfordMA, USA

**Keywords:** load theory, executive capacity, ERPs, selective attention, working memory

## Abstract

The classic account of the load theory (LT) of attention suggests that increasing cognitive load leads to greater processing of task-irrelevant stimuli due to competition for limited executive resource that reduces the ability to actively maintain current processing priorities. Studies testing this hypothesis have yielded widely divergent outcomes. The inconsistent results may, in part, be related to variability in executive capacity (EC) and task difficulty across subjects in different studies. Here, we used a cross-modal paradigm to investigate whether augmented working memory (WM) load leads to increased early distracter processing, and controlled for the potential confounders of EC and task difficulty. Twenty-three young subjects were engaged in a primary visual WM task, under high and low load conditions, while instructed to ignore irrelevant auditory stimuli. Demands of the high load condition were individually titrated to make task difficulty comparable across subjects with differing EC. Event-related potentials (ERPs) were used to measure neural activity in response to stimuli presented in both the task relevant modality (visual) and task-irrelevant modality (auditory). Behavioral results indicate that the load manipulation and titration procedure of the primary visual task were successful. ERPs demonstrated that in response to visual target stimuli, there was a load-related increase in the posterior slow wave, an index of sustained attention and effort. Importantly, under high load, there was a decrease of the auditory N1 in response to distracters, a marker of early auditory processing. These results suggest that increased WM load is associated with enhanced attentional engagement and protection from distraction in a cross-modal setting, even after controlling for task difficulty and EC. Our findings challenge the classic LT and offer support for alternative models.

## Introduction

Selective attention reflects a set of cognitive processes that filters incoming information to maintain ongoing cognitive activity (Broadbent, 1954), allowing individuals to focus on what is important while ignoring irrelevant information. The load theory (LT) of selective attention, which has elicited a spirited debate in the literature, proposes that two fundamental mechanisms mediate selective attention. The first mechanism is perceptual, excluding distracters from perception when the level of perceptual load in processing task-relevant stimuli is high enough to exhaust perceptual capacity. This is a passive selection process that occurs because under high perceptual load, limited resources are available to process distracters. By contrast, in situations of low perceptual load, spare capacity not used to process task-pertinent stimuli will “spill over” to the processing of irrelevant distracters (Lavie and Tsal, 1994; Lavie, 1995; Lavie and Cox, 1997; Rees et al., 1997; Lavie et al., 2014). The second mechanism reflects “active” cognitive control that depends on higher cognitive functions, such as the executive control component of working memory (WM) (Lavie et al., 2004; Lavie, 2005). In contrast to perceptual load, distracter interference increases under high WM load. Carrying out the more demanding primary task is associated with augmented competition for the pool of limited executive control resources, resulting in the reduced ability of individuals to actively maintain current processing priorities that distinguish between relevant and irrelevant information (de Fockert et al., 2001; Lavie et al., 2004; Lavie and De Fockert, 2005; Rissman et al., 2009; Burnham, 2010; Kelley and Lavie, 2011).

Studies that have tested the cognitive load aspect of LT have yielded very inconsistent findings. For example, there is behavioral and neurophysiological data to suggest that increasing demands on the executive control component of WM lead to an increase in distracter effects, in keeping with the tenets of LT (Sreenivasan et al., 2007; Konstantinou et al., 2012, 2014; Konstantinou and Lavie, 2013; Roper and Vecera, 2014). In stark contrast, other studies have demonstrated a reduction in the processing of task-irrelevant distracters when demands on executive control are augmented, as indexed by decreased amplitudes of event related potentials (ERPs), such as N1, P3, and reorienting negativity (RON) (Berti and Schroger, 2003; Rose et al., 2005). These findings are not consistent with LT. Similarly, neuroimaging data suggest that increased cognitive demands (e.g., mental subtractions) are associated with a reduction of activity in visual areas related to the processing of distracters (Spinks et al., 2004).

Working memory load has also been investigated in cross-modal studies. It is worth noting that in many real world situations, individuals must contend with multimodal stimulation (e.g., reading a newspaper or driving while ignoring irrelevant sounds). The investigation of the impact of cognitive WM load on cross-modal processing has also produced mixed results. For instance, some studies suggest that cognitive WM load does not influence early processing of task-irrelevant auditory stimuli, as indexed by the amplitude of N1 and mismatch negativity (MMN) components (Otten et al., 2000; SanMiguel et al., 2008). Other investigations have reported that increased WM load is associated with greater amplitude of the N1-P2 complex and MMN in response to task-irrelevant auditory stimuli, as would be predicted by LT (Zhang et al., 2006; Regenbogen et al., 2012). However, there is a body of evidence that has yielded the opposite findings, indicating that increasing WM load reduces the processing of task-irrelevant distracters in the auditory modality. For example, increasing WM load has been associated with a reduction of brainstem evoked responses to task-irrelevant sounds (Sörqvist et al., 2012a) and a decrease in the amplitude of different auditory ERPs, such as the MMN, P3, and RON (Munka and Berti, 2006; SanMiguel et al., 2008; Lv et al., 2010). Similarly, fMRI studies have found that under higher WM load, there is diminished activation in areas associated with processing of irrelevant stimuli presented in a different modality (Klemen et al., 2010; Regenbogen et al., 2012; Sörqvist et al., 2016).

Several hypotheses have been developed that may help account for the variability of findings in investigations of LT. For example, the absence of a clear operational definition of load in various studies could contribute to contradictory results (Benoni and Tsal, 2013). In many cases, increasing task demands may be associated with an augmentation of both perceptual and cognitive load, which according to LT would influence behavior and processing in opposing ways. In addition, because WM is not a unitary process (Smith and Jonides, 1999; Baddeley, 2012), manipulating the load on short-term maintenance operations may have a different impact on the processing of distracters than manipulating the demands on executive control operations, with the former being similar to augmenting perceptual load (Pasternak and Greenlee, 2005; Sreenivasan et al., 2007; Konstantinou and Lavie, 2013; Konstantinou et al., 2014; Roper and Vecera, 2014). Some research suggests that the impact of WM load on selective attention depends on the extent to which the contents of the WM task overlap with targets or distracters in a selective attention task (Yi et al., 2004; Kim et al., 2005; Park et al., 2007). Differences between experimental tasks also may contribute to conflicting evidence for LT. For example, according to Sörqvist et al. (2012b), when WM load is manipulated in a single-task paradigm (e.g., n-back task), processing of irrelevant information decreases under high load. However, processing of irrelevant information increases under high load when WM load is manipulated in a dual-task situation, such as maintaining items in WM while performing an unrelated task (see de Fockert et al., 2001; Lavie et al., 2004).

The current study focused on another potential source of the disparate findings associated with LT by considering the potential impact of individual differences in the executive capacity (EC) of subjects. Most investigations have not measured the WM capacity of subjects, nor accounted for the ways in which this factor may affect the relative difficulty of an experiment under different task loads. There is evidence that the same experimental task-load will be associated with different levels of difficulty depending on a subject’s WM capacity (de Fockert, 2013; Sörqvist and Rönnberg, 2014; Murphy et al., 2016). High WM capacity has been linked with a more steadfast locus of attention and less susceptibility to auditory distraction (Beaman, 2004; Sörqvist et al., 2012a,b; Hughes et al., 2013; Sörqvist and Rönnberg, 2014), a framework that we believe would benefit from further investigation using neurophysiological variables (e.g., ERP, fMRI).

Because predictions derived from LT are very dependent on the extent to which increased load taxes the WM capacity of subjects, thereby limiting available resources to maintain processing priorities, it is critical to attend to each subject’s individual capacity. Most studies in the LT field include small samples, which increases the likelihood of uneven distributions of the WM capacity of subjects across different investigations. For example, if a study were largely composed of subjects with low WM capacity, increasing task load for such individuals may severely strain available resources, making it very difficult for them to exert control over the processing of task-irrelevant distracters. In contrast, if another study mainly included subjects with high WM capacity, the same increase in task load would not deplete their resources. Rather, the more challenging task may further engage the attention of such subjects, resulting in diminished processing of distracters.

Sörqvist and Rönnberg’s recently developed the “neuro cognitive task-engagement/distraction trade-off” (TEDTOFF) model of WM capacity and cross-modal auditory distraction (Sörqvist and Rönnberg, 2014), which can help to scaffold the current study. The model assumes that task difficulty (e.g., load) and WM capacity (i.e., individual differences in the ability to control attention and filter task-irrelevant information) are critical factors that mediate distractibility. Within this framework, higher task difficulty leads to “protection” from distraction by increasing focal-task engagement (i.e., facilitating attention to the attended stimulus) and more actively suppressing the processing of irrelevant stimuli (Sörqvist and Marsh, 2015; Sörqvist et al., 2016). WM capacity is understood as reflecting individual differences in the ability of executive control operations to enhance the focus of attention on task-relevant stimuli and gate the processing of task-irrelevant stimuli. In contrast to LT, the TEDTOFF model considers the difficulty of the task to be more critical than the type of load manipulated (i.e., perceptual or cognitive) (Sörqvist and Rönnberg, 2014).

The aim of the present study was to investigate whether WM load modulates early distracter processing in a cross-modal paradigm. Subjects were engaged in a visual WM task (using an oddball paradigm) while instructed to ignore irrelevant auditory stimuli. Within this experimental context, we addressed some of the methodological limitations in the LT literature on cross-modal selective attention by controlling for the potential confounding effects of task difficulty and WM capacity. First, the EC of subjects, which incorporates indices of WM proficiency, was carefully measured using standard neuropsychological tests. This method has the advantage of relying on well-established norms to characterize the EC of our sample and facilitate comparisons with subjects who participate in future studies. Second, we titrated the level of difficulty of the high load condition so that it would have a relatively uniform impact on all participants. The low load condition was the same for all subjects, but in the high load condition, the number of visual targets was determined individually for each participant to keep task performance consistent across subjects (i.e., accuracy rate of ∼80%). In accordance with our framework, we anticipated that subjects with higher EC would have to be given a larger number of target stimuli to hold in WM to make their performance comparable to that of subjects with lower EC. An important measure of the success of our titration process would be finding that under the high WM load condition, performance on the primary task did not vary as a function of EC or the number of target letters presented to subjects. To the best of our knowledge, no prior study of LT has controlled for variation in subjective difficulty of the primary task across participants. This approach allowed us to address the impact of increased cognitive load of the visual task on the processing of task-irrelevant auditory stimuli in a manner that limited the influence of differences in subjective task difficulty and EC across subjects.

ERPs were used to measure neural activity in response to stimuli presented in both the task relevant modality (visual) and task-irrelevant modality (auditory). ERPs in response to stimuli from the primary task provided neural indices of attentional engagement and resource utilization under different load conditions. The posterior slow wave (SW) served as an index of sustained attention and effort (Ruchkin et al., 1980; Gevins et al., 1996; Rushby et al., 2005; Daffner et al., 2011). We anticipated that in response to visual targets, the posterior SW would increase under the high load condition (Ruchkin et al., 1980; Ruchkin and Sutton, 1983). Also, the P3b component was measured as an index of the process of categorizing an event (Squires et al., 1973; Ford, 1978; Kok, 2001) or updating memory after an event has been categorized (Donchin, 1981; Donchin and Coles, 1988). Consistent with many reports in the literature, we expected a decline in the visual P3 amplitude under the high load condition (Mecklinger et al., 1992; Ruchkin et al., 1992; Gevins et al., 1996). It has been hypothesized that resources under the low load condition had been devoted to decision-making/updating are “reallocated” under the high load condition to the operations of maintenance, manipulation, and sustaining attention, leading to a decline in P3 amplitude (McEvoy et al., 1998; Watter et al., 2001; Daffner et al., 2011). The auditory N1 component, a frontocentral negativity peaking 50–150 ms after stimulus onset, served as a measure of early perceptual processing of task-irrelevant auditory distracters (Woods, 1995). Our primary test of LT involved determining the impact of visual WM load on the amplitude of the auditory N1. If we found that WM load of the primary visual task had no effect on the N1 response to task-irrelevant auditory stimuli, it would be consistent with the idea of separate pools of attentional resources within each sensory modality that do not interfere with each other (Otten et al., 2000). In contrast, finding that visual WM load modulates N1 response in the unattended auditory modality would suggest a shared reservoir of resources, with potential for interference between modalities. Observing that increasing WM load in the primary task is associated with greater processing of distracter stimuli (as indexed by an enhanced auditory N1) would be consistent with the classic LT of cognitive control. In contrast, if we found that augmenting WM load is coupled with an attenuation of the auditory N1, our results would be a challenge to LT. Such findings would be more consistent with the TEDTOFF model that suggests that higher visual WM load increases attentional engagement and “protects” from distraction.

## Materials and Methods

### Participants

Subjects were recruited through community announcements in the Boston metropolitan area. A total of 23 subjects (age range 19–30 years) participated in the study. An additional 6 subjects completed the experiment, but were excluded due to excessively noisy data (20% or more epochs rejected by the automated artifact rejection software). The research protocol was approved by the Human Research Committee for the Partners Healthcare System and was conducted according to the principles expressed in the Declaration of Helsinki. All participants provided written informed consent.

In the initial session, all subjects completed a detailed screening evaluation that included a structured interview to obtain a medical, neurological, and psychiatric history; a formal neurological examination, audiologic evaluation, test of visual acuity (via Snellen Wall chart); and the completion of a neuropsychological test battery, questionnaires surveying mood and daily living activities, and completion of the titration procedure to determine number of targets under the high load condition (see below). In a subsequent visit, subjects participated in the experimental protocol. Subjects were paid for their time.

To be included in the study, participants had to be 18–35 years old, English-speaking, and have ≥12 years of education, an estimated intelligence quotient (IQ) ≥100 on the American National Adult Reading Test (AMNART) (Bright et al., 2002). Subjects were excluded if their mean percentile performance relative to age-appropriate norms across selected neuropsychological tests (described below) was in the bottom third (<33rd percentile)^1^. Participants were also excluded if they had a history of CNS diseases or major ongoing psychiatric disorders based on DSM-IV criteria (American Psychiatric Association, 1994), focal abnormalities on neurological examination consistent with a CNS lesion, a history of clinically significant medical diseases, or corrected visual acuity worse than 20/40. Moreover, subjects underwent pure tone audiometry in which hearing thresholds were tested at 250, 500, 1000, 2000, and 4000 Hz, and excluded if they demonstrated the following abnormalities: >40 dB mean loss across frequencies, >20 dB difference between ears at any frequency, or >30 dB difference between the best and worst threshold (Friedman et al., 1998). No subjects were excluded on the basis of impairments in vision or hearing.

In measuring EC, we followed the suggestion of many investigators who emphasize processes that include WM, initiation, monitoring, and inhibition, and advocate the use of several neuropsychological tests to assess this complex group of functions (Spreen and Strauss, 1998; Delis et al., 2001; Lezak et al., 2004; Chan et al., 2008). Tests included: (1) Digit Span Backward subtest of the Wechsler Adult Intelligence Scale-IV (WAIS-IV) (Wechsler, 2008), which measures maintenance and manipulation operations of WM; (2) Letter–Number Sequencing subtest (WAIS-IV), which assesses monitoring, inhibition, and manipulation; (3) Digit–Symbol Coding subtest (WAIS-IV), which assesses sustained attention, cognitive speed, and inhibition; (4) Controlled Oral Word Association Test (COWAT) (Ivnik et al., 1996), which indexes initiation, self-generation, and monitoring; (5) Trail-Making Test Parts A and B (Reitan and Wolfson, 1985), which measure planning/sequencing, set shifting, and inhibition. Subjects were divided into two EC groups: high EC subjects were those whose mean percentile score based on age-appropriate norms was >66.6; average EC subjects had a mean percentile score based on age-appropriate norms of 33.3–66.6.

### Experimental Procedure

**FIGURE 1:**
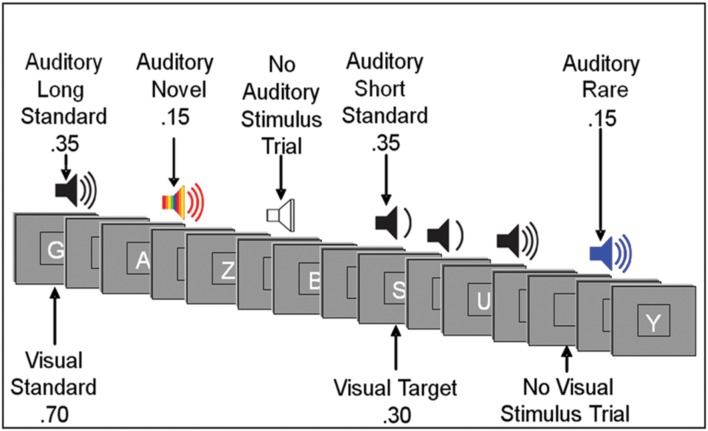
**Illustration of an experimental run.** Subjects responded to visual target letters (forced choice) while instructed to ignore auditory stimuli. Visual stimuli were presented for 200 ms. Auditory standard and rare stimuli were presented for 250 ms or 125 ms; auditory novel stimuli were presented for 250 ms. The interstimulus interval varied randomly between 315 and 665 ms. (See text for more details.).

Event-related potentials were recorded during a forced-choice visual oddball paradigm. Subjects were instructed to respond to target stimuli and non-target stimuli with opposite mouse clicks, i.e., left click for target stimuli (i.e., specific letter(s) predefined as a target) and right click for non-target stimuli. The hand used for the target response was counterbalanced across subjects. The subjects were also instructed to ignore sounds. Order of stimuli presentation varied randomly across blocks within the visual task, and across conditions. Presentation of letters and sounds did not temporally overlap (see **Figure 1**).

The task included 800 stimulus trials divided into 8 blocks, separated into 4-block sections for low and high WM load. Under the low load task, subjects were required to keep one visual target letter in mind. Under the high load task, the number of unique target letters required was determined by subject performance on a titration task. During the titration task, subjects were tested on consecutive blocks of the visual task without auditory stimuli. The number of unique letters designated as target stimuli was gradually increased across blocks. The number of target letters for which subjects scored closest to 80% accuracy (target hit ratio – false alarm ratio) was chosen to be used for high visual task load condition.

Visual stimuli appeared one at a time within a fixation box that remained on the screen at all times and subtended a visual angle of ∼3.5° × 3.5° at the center of a high-resolution computer monitor. Visual stimuli subtended an angle of 2.5° along their longest dimension and were presented for 200 ms. Target letters comprised 30% of visual stimuli. Non-target letters comprised 70% of visual stimuli. Auditory stimuli were presented one at a time with a minimum intensity of 75dB SPL. Standard auditory stimuli, comprising 70% of auditory stimuli, were 250 Hz pure tones presented for a duration of either 250 ms (35%) or 125 ms (35%). Rare auditory stimuli, comprising 15% of auditory stimuli, were 500 Hz pure tones of either long (250 ms) or short (125 ms) duration. Short and long rare stimuli were not presented in equal proportion: each comprised 80 or 20% of total rare auditory stimuli and were counterbalanced across subjects. Novel auditory stimuli were complex, environmentally derived or synthesized sounds presented for a duration of 250 ms, comprising 15% of auditory stimuli. Each novel auditory stimulus was unique within and between tasks. Due to our interest in the early processing of task-irrelevant auditory distracters, the current study focused on the auditory N1 component, which is commonly investigated in response to standard stimuli (Näätänen and Picton, 1987; Munte et al., 1998; Schroder et al., 2014). The electrophysiologic response to other classes of stimuli (e.g., novel, rare) will be the subject of future reports.

Auditory stimuli had ∼20 ms rise/fall times. The inter-stimulus interval (ISI) between auditory and visual stimuli varied randomly between 315 and 665 ms (mean ∼490 ms). In addition to the 400 visual and 400 auditory stimulus trials, there were 100 auditory and 100 visual trials devoid of a stimulus. For visual stimuli, a no-stimulus trial appeared as a blank presentation box. For auditory stimuli, a no-stimulus trial was a period of silence when an auditory stimulus was to be expected.

### ERP Recordings

An ActiveTwo electrode cap (Behavioral Brain Sciences Center, Birmingham, UK) was used to hold to the scalp a full array of 128 Ag-AgCl BioSemi (Amsterdam, The Netherlands) “active” electrodes whose locations were based on a pre-configured montage. Electrodes were arranged in equidistant concentric circles from 10–20 system position Cz. In addition to the 128 electrodes on the scalp, 6 mini bio-potential electrodes were placed over the left and right mastoid, beneath each eye, and next to the outer canthi of the eyes to check for eye blinks and vertical and horizontal eye movements. EEG activity was digitized at a sampling rate of 512 Hz.

### Data Analysis

#### Behavioral Data

Demographic variables and neuropsychological test performance of the high vs. average EC groups were compared used independent sample *t*-tests. Mean target accuracy and mean reaction time (RT) during the visual task were also measured. Due to technical issues during recording, behavioral data are missing for two subjects. A correct response was considered a hit if it occurred between 200 and 1000 ms after stimulus presentation. Ratios of target stimuli correctly responded to (target hits) and stimuli incorrectly identified as targets (false alarms) were calculated in order to determine an overall accuracy score (percent target hits minus percent false alarms).

#### ERP Data

EEG data were analyzed using ERPLAB (Lopez-Calderon and Luck, 2014) and EEGLAB (Delorme and Makeig, 2004) toolboxes that operate within the MATLAB framework. Raw EEG data were resampled to 256 Hz and referenced off-line to the algebraic average of the right and left mastoids. EEG signals were filtered using an IIR bandpass filter with a bandwidth of 0.03–40 Hz (12 dB/octave roll-off for all). Eye artifacts were removed through independent component analysis after visual inspection of the generated components. Individual channels that revealed, upon visual inspection, a consistently different pattern of activity from surrounding channels were corrected with the EEGLAB interpolation function. The sampling epoch for each trial lasted for 1200 ms, including a 200 ms pre-stimulus period that was used to baseline correct the ERP epochs. Trials were discarded from the analyses if they contained baseline drift or movement artifacts greater than 90 μV. Only trials with correct responses were included in the analyses.

#### Average Waveform Analysis

Event-related potentials in response to auditory standard stimuli and visual target stimuli were measured at regions of interest (ROIs). ROIs were selected based on topographic scalp plots of ERP components. The analyses focused on the posterior SW and visual P3b in response to visual target stimuli and auditory N1 in response to auditory standard stimuli. For the visual P3b, mean amplitude was measured over the 100 ms window centered at individual peak latencies for each load in each EC group, based on statistical differences in local peak latency. Mean amplitude of the visual SW was measured from 600 to 1000 ms based on visual inspection of the waveform. For N1, mean amplitude was measured over the 40 ms window centered on the grand mean local peak latency. (see **Table 1**).

**Table 1:** Regions of interest (ROIs) and time windows selected to calculate the amplitudes of the ERPs components.

Auditory Component		Visual Components	
	
	N1	P3	Slow wave (SW)
Site	Frontal central	Posterior central	Posterior central
	ROI	ROI	ROI
Time window (ms)	96–136	HEC, LL 357–457	600–1000


		HEC; HL: 473–573	
		AvEC, LL: 401–501	
		AvEC, HL: 492–592	


*AvEC = Average EC; HEC = High Executive Capacity; HL = High Load.*

*LL = Low Load; ROI = Region of Interest.*

#### Temporospatial Principal Component Analysis (PCA)

To further validate the findings from the average waveforms, we also performed a temporospatial principal component analysis (PCA), a data–driven method that decomposes ERP waveforms into their underlying components and is particularly useful in parsing spatially and temporally overlapping components (Dien, 2012). Following the recommendation of Dien (2012) and our past work (Alperin et al., 2013, 2014; Porto et al., 2015) the temporospatial PCA (temporal PCA followed by spatial PCA) was conducted on averaged trials for each individual subject at all 134 electrode sites. Two separate PCAs were conducted for auditory and visual stimuli.

Utilizing the ERP PCA toolkit 2.38 (Dien, 2010), Promax and Infomax rotations were used for temporal and spatial PCAs, respectively, and a covariance matrix and Kaiser normalization were applied to the data. Each dataset consisted of 347 time points between -200 and 1000 ms. A parallel test was used to restrict the number of factors generated for each PCA. Consistent with the literature, factors of interest were selected based on visual inspection of the timing and topography of the output (Spencer et al., 1999; Goldstein et al., 2002; Dien et al., 2003). Any factors that accounted for >2% of the total variance were considered for further analyses (Dien, 2012).

### Statistical Analysis

Comparisons between EC groups for demographic and neuropsychological variables were carried out using *t*-tests for continuous variables, and the chi-squared test for categorical variables. In addition, a 2 × 2 analysis of variance (ANOVA), including load as a within subject factor (high vs. low) and EC group as between subjects factor (high vs. average) was used to analyze the behavioral data and the ERP components (i.e., posterior SW, P3b, and N1). A *p* < 0.05 was considered significant. Statistical analyses were performed using the Statistical Package for Social Sciences (SPSS) version 23.

## Results

### Participants

**Table 2** summarizes subject characteristics, including demo graphic and neuropsychological data. There was no difference between the two EC groups in terms of age [*t*(21) = 0.53 *p* > 0.5], sex [*x*^2^ = 0.05 *p* = 0.83], and estimated IQ [*t*(21) = 1.96 *p* > 0.05]. Years of education were lower in the average EC group [*t*(21) = 3.44 *p* < 0.01]. As expected, individuals classified as high EC performed better on tests of executive functions, with higher EC percentile score [*t*(21) = 7.43 *p* < 0.001]. Of note, subjects with higher EC scored much better on all but one of the cognitive tests (Digit Symbol, which did not reach significance).

**Table 2:** Demographic and neuropsychological characteristics of each Executive Capacity (EC) group.

	High EC *M* (*SD*)	Average EC *M* (*SD*)	*p*-value
N	12	11	-
Sex (F:M)	6:6	6:5	0.83
Age (years)	23.1 (2.6)	22.5 (2.8)	0.56
Education (years)	16.1 (1.3)	14.1 (1.3)	0.002
IQ - AMNART	120.0 (5.2)	114.9 (7.3)	0.06
EC Percentile Score	80.3 (7.2)	54.1 (9.5)	<0.001
Targets under high load^a^	8.2 (1.0)	6.9 (0.8)	0.003


*AMNART, American version of the National Adult Reading Test; EC, Executive. Capacity; M, Mean; N, Number; SD, Standard Deviation; ^a^Number of target letters presented under the high load condition.*

### Behavioral Data

#### Titration and Performance

As noted, the titration procedure aimed for an accuracy rate of ∼80% in each subject. The number of visual targets presented under the high load varied across EC groups [*t*(21) = 3.36 *p* = 0.003], with the high EC group being presented with a greater number of visual targets than average EC group (**Table 2**).

There was an effect of load on the primary task’s accuracy [*F*(1,19) = 7.51 *p* = 0.01] and RT [*F*(1,19) = 118.15 *p* < 0.001]. As illustrated in **Table 3**, accuracy was lower and RTs were longer under high than low WM load, indicating that the high load task was more difficult to perform. There was no effect of EC group on accuracy [*p* = 0.30] or RT [*p* = 0.54], and no interaction was observed between load and EC group for accuracy [*p* = 0.46] or RT [*p* = 0.29].

**Table 3:** Behavioral performance on primary task (visual).

	High EC (*N* = 10) *M* (*SD*)	Average EC (*N* = 11) *M* (*SD*)	*p*-value
Accuracy low load	0.79 (0.1)	0.86 (0.1)	0.45
Accuracy high load	0.76 (0.1)	0.79 (0.1)	0.25
RT low load (ms)	477.0 (33.6)	501.7 (56.3)	0.24
RT high load (ms)	600.3 (74.4)	603.0 (52.6)	0.92


*EC, Executive Capacity; M, Mean; RT, Reaction Time; SD, Standard Deviation.*

### Event Related Potentials^2^

#### Visual Target Stimuli in Primary Task

##### Average waveform analysis

**FIGURE 2:**
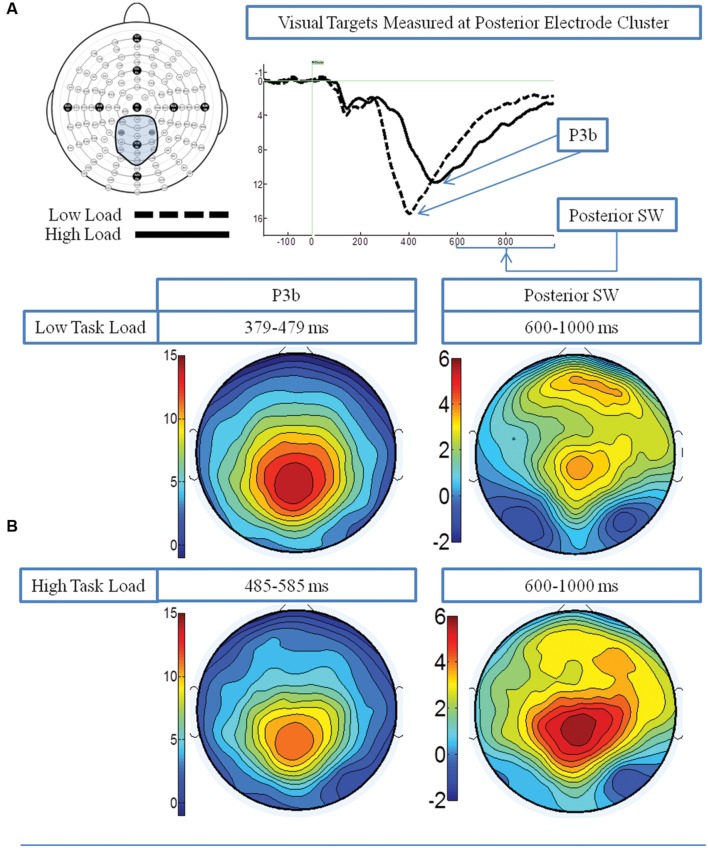
**Event-related potential (ERP) data in response to target visual stimuli under the low and high load conditions.** **(A)** Illustrates the grand average waveforms (arrows point to the P3b component and posterior SW); and **(B)** shows the surface potential maps for the P3b component and the posterior SW.

**Figure 2A** presents the grand average waveforms in response to target stimuli under low and high load conditions at specified ROIs, and **Figure 2B** illustrates the surface potential maps of the posterior SW and P3b.

**FIGURE 3:**
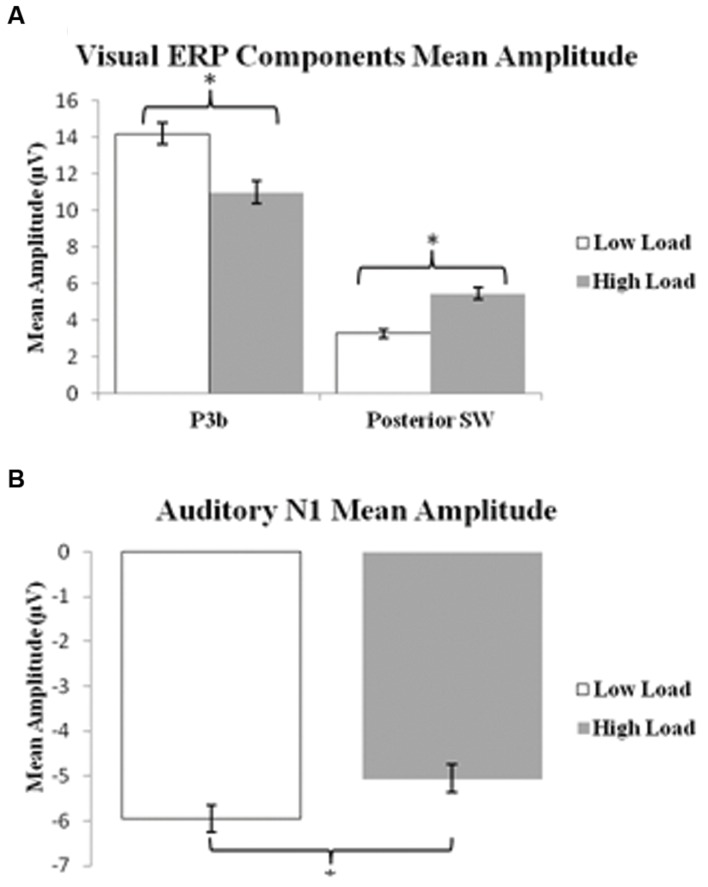
**Bar graphs illustrating the mean amplitudes of the ERP components under the low and high load conditions.** **(A)** Shows mean amplitude of P3b component and posterior SW in response to target visual stimuli; and **(B)** illustrates mean amplitude of the N1 component in response to standard auditory stimuli. Error bars indicate standard error of the mean. ^∗^*p* < 0.05.

P3b: A 2 load × 2 EC group ANOVA revealed an effect of load on P3b amplitude [*F*(1,21) = 13.96 *p* = 0.001], no effect of EC group [*p* = 0.16], and no interaction between load and EC group [*p* = 0.62]. The load effect was present because the P3b amplitude was smaller under the high than low load condition, as illustrated in **Figure 3A.**

Posterior SW: The analysis showed an effect of load on posterior SW amplitude [*F*(1,21) = 18.72 *p* < 0.001], which was greater under the high load condition (**Figure 3A**). There was no effect of EC group [*p* = 0.36]. There was a trend toward an interaction between load and EC group [*p* = 0.07], which was due to the magnitude of the load effect being larger for the high EC group.

##### PCA analysis

**FIGURE 4:**
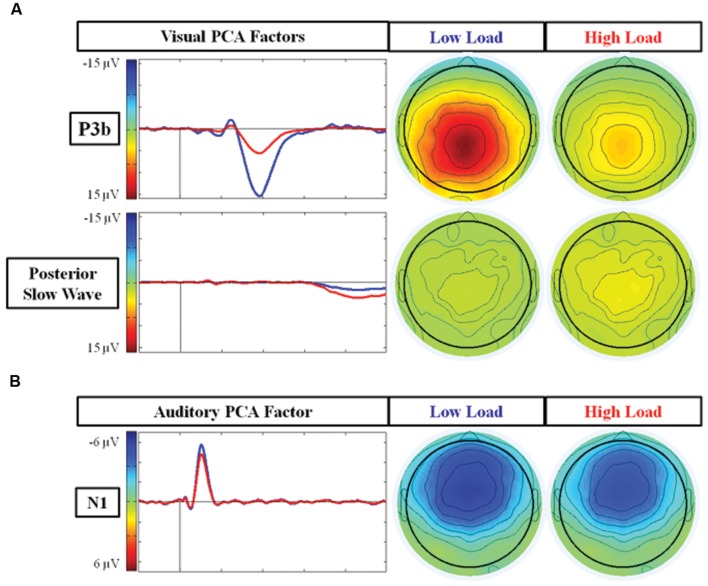
**Scalp topographies and waveforms of PCA factors under the low and high load conditions.** **(A)** Illustrates the PCA factors reflecting the P3b component (TF2SF1) and posterior SW (TF3SF1); and **(B)** represents the PCA factor reflecting the N1 component (TF4SF1). TF, Temporal Factor. SF, Spatial Factor.

**Figure 4A** illustrates the two PCA factors in response to target stimuli that correspond to the P3b and posterior SW. Based on temporal course and spatial distribution, one temporospatial PCA factor (TF2SF1) was identified as reflecting the P3b, peaking at electrode site A4 (near Pz) at 386 ms, accounting for 21.1% of total variance. Similarly, based on its temporal course and spatial distribution, one temporospatial PCA factor (TF3SF1) was identified as reflecting the posterior SW, peaking at electrode site C2 (near Cz) at 863 ms, accounting for 8.5% of total variance.

There was an effect of load for the factor representing the P3b component (TF2SF1) [*F*(1,21) = 74.97 *p* < 0.001], and for the factor representing the posterior SW component (TF3SF1) [*F*(1,21) = 5.75 *p* = 0.02]. There was no effect of EC group [*p*s > 0.15] nor interaction between load and EC group for either of these factors [*p*s > 0.4]. For the P3b component, the factor score (amplitude) was larger under the low load than the high load, whereas for the posterior SW component, the factor score was larger under high than low load. In summary, the main results of the PCA were consistent with those found of the average waveforms. However, in contrast to the average waveform analysis, there was no trend toward an interaction between load and EC for the PCA factor reflecting the posterior SW.

#### N1: Cross-modal Effect

##### Average waveform analysis

**FIGURE 5:**
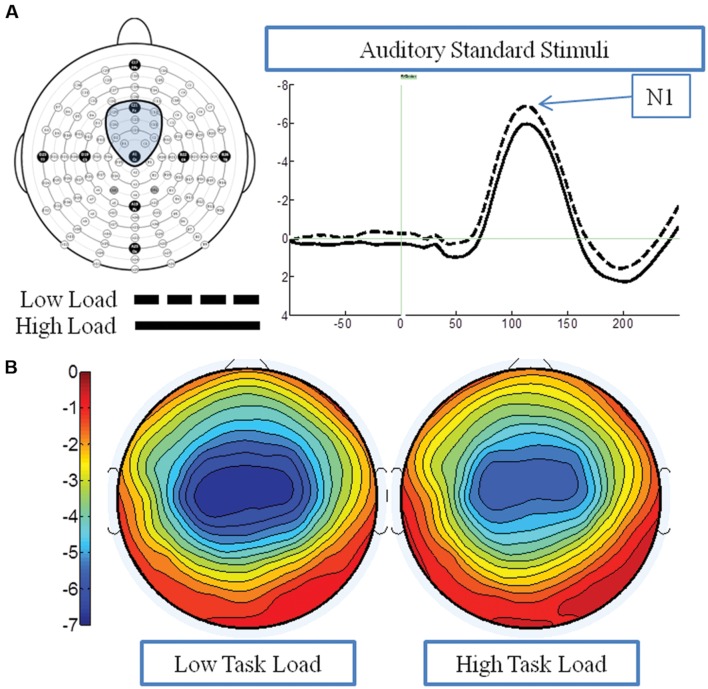
**Event-related potential data in response to standard auditory stimuli under the low and high load conditions.** **(A)** Illustrates the grand average waveform (arrow points to the N1 component); and **(B)** shows the surface potential maps for the N1 component.

**Figure 5A** presents the grand average waveforms in response to auditory stimuli under low and high load conditions at specified ROIs, and **Figure 5B** illustrates the surface potential maps of the posterior N1. An ANOVA revealed an effect of load for the N1 component to auditory standards, with a decrease of N1 amplitude under high load [*F*(1,21) = 8.54 *p* = 0.006] (**Figure 3B**). There was no effect of EC, nor interaction between EC group and load [*p*s > 4].

##### PCA analysis

**Figure 4B** illustrates the temporospatial PCA factor (TF4SF1) that reflects the N1 to auditory standard stimuli based on its frontal distribution and latency. It peaked at electrode site FCz at 105 ms and accounted for 5.0% of total variance. The ANOVA for the PCA factor reflecting the N1 confirmed the average waveform analysis: there was an effect of load [*F*(1,21) = 7.71 *p* = 0.01], with smaller response under high load. There was no effect of EC group [*p* = 0.56] nor interaction between load and EC group [*p* = 0.61].

##### Correlational analyses

Correlational analyses using Spearman’s rho were run to explore the relationships between pertinent ERP components and behavioral factors. The focus was on load-related differences in the variables. Our most salient findings include the following: (a) the larger the load-related increase in posterior SW to visual targets, the larger the load-related decrease in N1 to auditory standards [ρ = 0.54, *p* = 0.008]; (b) the larger the load-related reduction in N1 to auditory standards, the smaller the load-related decrease in visual target accuracy [ρ = 0.52, *p* = 0.01]; (c) the larger the load-related increase in posterior SW to targets, the smaller the load-related decrease in accuracy to visual targets [ρ = 0.53, *p* = 0.01]; (d) the smaller the load-related decrease in P3b to visual targets, the smaller the load-related increase in RT to visual targets [ρ = -0.47, *p* = 0.03]; and (e) the smaller the load-related increase in RT, the smaller the load-related decrease in accuracy [ρ = -0.37, *p* = < 0.001]. Also of note, there was no correlation between performance on the visual task (i.e., accuracy and RT) under the high load condition, and the number of target letters presented under the high load condition [*ps* > 0.65].

##### Additional analyses

A potential explanation for the load-related decrease in the N1 amplitude is that it is a direct reflection of the impact of the load-related increase in the posterior SW, which overlaps temporally with the subsequent presentation of auditory stimuli (see **Figure 1**). Within this framework, the two components would be the result of the same effect, time-locked to different experimental stimuli. For this account to be plausible, there must be not only a temporal, but also a spatial overlap between these two components. Of note, there is no spatial intersection between the electrode cluster used to measure the posterior SW and the cluster used to measure the N1 component. However, this does not preclude the possibility that the electrophysiologic activity of the posterior SW extends to sites more anterior than those included in the measurement of the posterior SW. To address this issue, SW activity in response to visual target stimuli was re-measured at the Fz electrode cluster (used to measure N1 amplitude) to determine if there was an effect of load. Results show that the amplitude of the SW at the Fz cluster did not differ across loads [*p* > 0.07]. Also, the load-related change in the SW at the Fz cluster did not correlate with the load-related change of the N1 (*p* > 0.17). Moreover, if there were a direct link between the posterior SW to visual targets and the N1 to subsequent auditory standards, one would anticipate that within each load there would be a strong inverse correlation between the two components, which was not observed [*ps* > 0.4]. Nor was there an inverse correlation between the amplitude of the SW measured at the Fz cluster and the N1 measured at the same location.

As discussed in the Introduction, one hypothesis for the decline in the N1 amplitude under the high load condition is that it reflects a passive process (i.e., depletion of available resources due to augmented processing of the primary task, as measured by the increase in the posterior SW). If this were the case, one would expect to also find an inverse correlation between the amplitude of the N1 to auditory distracters and the amplitude of the posterior SW to visual targets when analyzed for each load separately, which, as noted, was not found. Taken together, these results suggest that the load-related decrease in N1 amplitude may reflect a more active, top–down process, rather than a passive process.

## Discussion

The present study employed a cross-modal paradigm to investigate the impact of WM load on distracter processing, using ERPs as critical dependent variables. To address a potential source of inconsistent findings in the LT literature, the study aimed to control for the confounding effects of task difficulty and EC across subjects. Well-normed, standardized neuropsychological tests rather than experimental tasks were used to define EC, which may allow for greater generalization of results. The WM load manipulation of the primary visual task was effective: relative to the low load condition, the high load condition was associated with lower accuracy and longer RTs. The success of the titration process in controlling the level of task difficulty across subjects was demonstrated by finding that under high WM load, behavioral performance on the primary task was not modulated by EC group. As expected, to achieve this goal, under the more demanding load condition, subjects with higher EC needed to be given a significantly greater number of target stimuli to make their performance comparable to that of subjects with lower EC. Similarly, the ERP indices of processing task-irrelevant stimuli were not modulated by EC group. A trend was noted in the average waveforms suggesting a greater load-related augmentation of posterior SW activity to visual targets for the high than average EC group. This finding may indicate that in response to a similar level of task difficulty, subjects with higher EC are able to mobilize more attentional resources, which would be consistent with the view that higher WM capacity is associated with a more steadfast locus of attention (Sörqvist and Rönnberg, 2014; Sörqvist and Marsh, 2015).

The N1 component served as a marker of early processing of task-irrelevant auditory distracters. The tenants of LT lead to the prediction that augmenting WM load of the primary visual task would result in an increase in the N1 amplitude to auditory distracters because competition for limited EC resources reduce an individual’s ability to sustain current processing priorities. The main findings of the experiment are not consistent with LT. Increasing WM load was linked to a decrease, not an increase, in N1 amplitude in response to task-irrelevant auditory distracters. Our results are consistent with previous findings indicating that augmentation of WM load is associated with a reduction in the magnitude of auditory-evoked brainstem potentials to irrelevant sounds, a marker of the very earliest processing of auditory distracters (Sörqvist et al., 2012b). In line with our findings, other groups have also reported reduced distracter processing under high compared to low WM load in cross-modal paradigms (Munka and Berti, 2006; SanMiguel et al., 2008; Klemen et al., 2010; Regenbogen et al., 2012; Sörqvist et al., 2012a, 2016). Importantly, this pattern of findings was upheld after controlling for the potential influence of differences in subjective task difficulty and EC across participants.

The results of the current study are in line with the TEDTOFF model, which states that augmenting task difficulty (i.e., WM load) is associated with increased attentional engagement and “protection” from distraction (Sörqvist and Rönnberg, 2014; Sörqvist, 2015, Sörqvist et al., 2016). Our ERP data offer additional support for this theory by providing a non-behavioral measure of increased “steadfastness” of the locus of attention. Our results demonstrated that the amplitude of the posterior SW increased under the high load condition, consistent with previous work (Ruchkin et al., 1980; Ruchkin and Sutton, 1983; Daffner et al., 2011). There is evidence that the posterior SW is an index of sustained attention and effort processes that likely underlie the steadfastness of attention (Ruchkin et al., 1980; Gevins et al., 1996; Rushby et al., 2005). Correlation analyses indicated that when WM load is augmented, the greater the increase in attention to the primary task (as measured by the posterior SW), the greater the reduction of the processing of task-irrelevant auditory standards (as measured by the N1). Moreover, as WM load increases, a smaller decline in target accuracy is associated with ERP measures of enhanced attention to the primary task (indexed by the posterior SW) and reduced processing of irrelevant stimuli (indexed by the N1).

One concern is that the load–related increase in posterior SW to visual targets directly influences the measurement of the subsequent N1 to auditory standards, reflecting the same effect that is time-locked to different experimental stimuli. This account could explain why the load–related differences in the average waveforms to auditory standards appear to begin prior to the N1 itself. There are several reasons why we believe this explanation to be unlikely. First, the interstimulus interval (ISI) between visual and auditory events was jittered between 315 and 665 ms, reducing the likelihood that electrophysiologic responses to auditory events were time-locked to visual events (Luck, 2005). Second, there was limited overlap in the spatial distribution of the posterior SW to visual targets and N1 to auditory standards, which would be necessary to directly link the measurement of one component to that of the other. Also, when the SW was re-measured at the Fz cluster (where the N1 was measured) no load effect was observed. Moreover, if the posterior SW and N1 reflected the same experimental effect, one would anticipate a close correlation between the two components within each load, but none was found. Finally, the pattern of results was confirmed using PCA, a method that can parse variance due to the N1 factor from that reflecting electrophysiologic activity that precedes or follows it (Dien, 2012). To further address this issue, future studies should include trials with longer ISIs to eliminate the possibility of temporal overlap between electrophysiologic responses to visual and auditory stimuli.

The dissociation found between the pattern of response for the posterior SW and the P3b component was predicted. In contrast to the SW, the amplitude of the P3b diminished under the high load condition, which is consistent with many other reports (Mecklinger et al., 1992; Ruchkin et al., 1992; Gevins et al., 1996). Under high load, resources that had been allocated to decision-making/updating may be utilized for other cognitive operations like sustaining attention, leading to greater uncertainty about target identity and a smaller P3b amplitude (DeSwart et al., 1981; Johnson, 1986; McEvoy et al., 1998; Watter et al., 2001; Daffner et al., 2011). Another potential contribution to load-related reduction of the size of P3b might be increased trial-to-trial variability in the peak latency of the P3b due to greater task difficulty. We tried to mitigate this potential confounder by measuring mean, and not peak, amplitude for the P3b, as previously recommended (Luck, 2005).

There are limitations to the current study, which focused on controlling for task difficulty and EC. It did not manipulate variables that have been shown to impact distracter processing using within-modality paradigms, such as which component of WM load is varied (e.g., maintenance vs. control), or the degree of overlap between the contents held in WM and the features of target or distracter stimuli (Kim et al., 2005; Park et al., 2007; Konstantinou et al., 2014), both factors that could be addressed in future research. Although our sample size was at least as large as many in the literature, it was nonetheless relatively modest and thus the findings need to be replicated. The inability to find a main effect of EC group or an interaction between EC group and load for our experimental variables could be the result of insufficient power. It is worth noting that almost none of the *p*-values from these analyses even approached a trend. Our analyses of the early processing of task-irrelevant auditory stimuli concentrated on standard stimuli, which is common to investigations that have relied on the N1 component (Näätänen and Picton, 1987; Munte et al., 1998; Schroder et al., 2014). Additional work is needed to determine whether a similar pattern of response would be observed for more salient events.

It would also be informative for future studies to include more challenging levels of WM load. We strongly suspect that the relationship between task difficulty and protection from distraction may follow an inverted U–shaped curve. If demands are sufficiently augmented, capacity–limited control resources would eventually be depleted, leading to an increase in the processing of task–irrelevant distracters. The theoretical curves of high EC individuals may be ‘shifted to the right’. If so, it would require much more demanding conditions for high EC adults to exhaust resources that mediate top–down control, resulting in an increase in distractibility. If this framework were correct, the TEDTOFF model, which is supported by the current findings, would require further modification. Finally, although we suspect that the correlation between the load-related increase in posterior SW to visual targets and the load-related decrease in N1 to auditory distracters is the result of active, top–down process and not a passive one (i.e., exhaustion of resources), additional research is necessary to confirm this hypothesis.

In summary, an increase in WM load was associated with electrophysiological evidence of an increased steadfastness of attention on the primary visual task and a reduction in the early processing of auditory distracters. These findings occurred within the context of controlling for the confounding effects of task difficulty and WM capacity across subjects, an approach that future studies should take into consideration. The results of this study present a challenge to LT, which may need to be modified.

## Author Contributions

KD and PH designed the experiment. ET analyzed the data and prepared the figures. KD, PH, ET, and SS interpreted the results of the experiment. SS drafted the manuscript. KD, PH, ET, and SS edited and revised the manuscript. KD, PH, ET, and SS approved the final version of the manuscript.

## Conflict of Interest Statement

The authors declare that the research was conducted in the absence of any commercial or financial relationships that could be construed as a potential conflict of interest.

## Footnotes

1 This criterion was adopted to exclude older adults with mild cognitive impairment in other experiments that compared subjects from different age groups. No older adults are included in the current study.

1 Because two subjects were missing from the behavioral results, data regarding subject characteristics and ERPs were re-analyzed for the remaining 21 subjects alone, which revealed the same statistical pattern as observed for the group of 23 subjects.
